# The relationship between placental thickness and gestational age in pregnant women: A cross‐sectional study

**DOI:** 10.1002/hsr2.1228

**Published:** 2023-04-27

**Authors:** Seyedeh H. Sharami, Forozan Milani, Sima Fallah Arzpeyma, Seyedeh F. Dalil Heirati, Zahra Pourhabibi

**Affiliations:** ^1^ Department of Obstetrics & Gynecology, Reproductive Health Research Center, Al‐zahra Hospital, School of Medicine Guilan University of Medical Sciences Rasht Iran; ^2^ Department of Radiology, Poursina Hospital, School of Medicine Guilan University of Medical Sciences Rasht Iran; ^3^ Reproductive Health Research Center, Al‐zahra Hospital Guilan University of Medical Sciences Rasht Iran; ^4^ Vice‐Chancellorship of Research and Technology Guilan University of Medical Science Rasht Zahra Iran

**Keywords:** GA, maternal BMI, placenta, placental thickness, posterior placental, pregnancy

## Abstract

**Background and Aims:**

Estimating gestational age (GA) is of utmost importance to assess the condition of the fetus. Incorporating placental thickness and fetal biometrics in estimating GA may improve the accuracy of fetal age estimation. The aim of this study was to examine the relationship between placental thickness and GA in pregnant women referred to Al‐Zahra Hospital's prenatal and emergency clinic in Rasht, Iran.

**Methods:**

This cross‐sectional study was conducted on pregnant women referred to Al‐Zahra Clinic for prenatal care over a 6‐month period. After obtaining informed consent, data were collected on the date of the first day of the last safe menstruation, average body mass index, and medical and surgical history. Placental thickness was estimated using ultrasound and various clinical information was recorded. The data were then analyzed using Pearson correlation analysis in SPSS software version 21.

**Results:**

The results showed a significant correlation between GA and placental thickness (*p* < 0.0001, *r* = 0.729). Placental thickness increased with increasing GA. There was also a significant relationship between placental thickness and placental location (*p* = 0.009, *r* = 0.14). In the posterior position, placental thickness increased by 14% or 0.14. The placental thickness in the posterior position (29.49 ± 0.75) was greater than the anterior position (26.94 ± 10.72).

**Conclusion:**

The findings of this study suggest that there is a significant increase in placental thickness with increasing GA during the first and second trimesters. Additionally, placental thickness significantly increased in the posterior placental position, as well as in women with high BMI. Therefore, it is recommended that measuring placental thickness should be routine during obstetric ultrasounds.

## 1 INTRODUCTION

In perinatal visits, accurate measurement of gestational age (GA) is critical for distinguishing between normal and abnormal pregnancy, timing screening tests, diagnosing intrauterine growth restriction (IUGR), and deciding on pregnancy termination. To determine GA and usual delivery time, the last menstrual period (LMP) is required. In such studies, the woman should have regular cycles and bleeding volume and not have used birth control tablets in the past 3 months.^1^, ^2^ Unfortunately, roughly 30% of patients do not meet these requirements, making estimated date of confinement (EDC) predictions based on LMP inaccurate. Ultrasound is currently the gold standard for determining GA by analyzing fetal dimensions such as crown‐rump length (CRL), biparietal diameter (BPD), head circumference (HC), abdominal circumference (AC), and femur length (FL). However, these criteria have limitations. For example, Biparietal diameter is not reliable for determining GA in babies with premature rupture of membranes, according to Wolfon et al. Therefore, other characteristics are required to estimate GA in fetuses with preterm rupture of membranes.^3^, ^4^

As a result, researchers are exploring other diagnostic markers, including thickness. The placental's appearance has received significant attention in recent years because of its connection to fetal growth and reflection fetal health. Mid‐pregnancy changes in the placenta, particularly between 17 and 20 weeks, have been shown to strongly correlate with fetal development and indicate fetal abnormalities.^5^, ^6^, ^7^

Ultrasound measurement of placental thickness is a simple and effective criterion for determining GA. The diameter around the head, biparietal diameter, abdominal circumference, and femoral length are all sonographically derived fetal characteristics that are used to date the pregnancy.^8^, ^9^ These are commonly used to estimate GA, either alone or in combination. Researchers have demonstrated that placental markers effectively assess children for GA (GA) and that intrauterine growth restriction (IUGR) requires early intervention.^10^, ^11^

Estimation of GA to determine placental thickness (PT), encourages obstetricians to regularly assess PT in pregnant women.^12^, ^13^, ^14^, ^15^ The combination of these measures with placental thickness may improve GA accuracy. Several studies have reported a relationship between placental thickness and GA in different weeks of pregnancy, but there is no consensus on the specific weeks.^12^, ^16^, ^17^ Furthermore, this relationship has varied across different breeds and placenta thickness ^16^ Given the importance of estimating GA for assessing fetal growth and development^7^, ^18^, ^19^; abnormal placental thickness has been widely investigated as a diagnostic criterion for evaluating pathological events,^20^, ^21^ but it has not been widely adopted in routine prenatal care. This study aimed examine the relationship between placental thickness and GA in pregnant women referred to the prenatal and emergency clinic at Al‐Zahra Hospita in Rasht, Iran.

## 2 PATIENTS AND METHODS

A cross‐sectional study was conducted using convenience sampling for a period of approximately 6 months at the prenatal clinic of Al‐Zahra Hospital in Rasht. Ethical clearance was obtained from the Guilan University Ethical Committee (IR.GUMS.REC.1397.93, and informed consent was obtained from all participants before the commencement of the study. After providing detailed information about the study and answering any questions that the female participants had, their written consent was obtained and they were enrolled in the study. The inclusion criteria included pregnant women with a GA of 11 weeks or more, aged between 20 and 35 years, without any complications, with a reliable date of the first day of the LMP, and no history of surgery or medical disease, and with a body mass index between 20 and 25. The exclusion criteria included patients with an unreliable LMP, chronic diseases such as diabetes, hypertension, or kidney disease, obesity (BMI above 30), multiple pregnancies, fetal‐placental abnormalities on ultrasound report, valentous or placental marginal fusion. Additionally, a difference of more than 4 weeks between amenorrhea and uterine height, or the presence of oligohydramnios, or polyhydramnios, were also exclusion criteria.

### 2.1 Scanning technique

Two collaborating radiologists evaluated all women with inclusion criteria using trans‐abdominal ultrasound at the Al‐Zahra Medical Training Center.

The evaluations included:

I. Fetal viability and detection of abnormalities.
II. Determination of GA.
III. Placental location.
IV. Grannum method of fetal placental grading.

Placental thickness was estimated using ultrasound in the sagittal view, starting from the umbilical cord entry site on the chorionic surface of the echogenic to the inner surface of the myometrial placenta. The closest point to the chorionic surface in the thickest part of the placenta, was considered the umbilical cord entry site, represented as a V‐shaped hypoechoic site. All placental parameters were measured during the uterine stasis phase, where the uterus was without contraction, as more placental thickness is reported during contractions. The researcher recorded the variables of age, weight, height, current GA, and obstetrical history (gravidity, parity).

### 2.2 Statistical analysis

All data were analyzed using the SPSS software package for Windows version 22.0 (SPSS Inc.). Mean and standard deviation, frequency, and percentage were used to describe the data. relationship between placental thickness and the studied variables was evaluated using Spearman correlation analysis. The significance level in this study was set at less than 0.05.

### 2.3 Ethical considerations

The study was approved by the Ethics Committee of Guilan University of Medical Sciences (IR.GUMS.REC.1397.93). All stages of the research were conducted according to the Helsinki Declaration. The procedures of the study were clearly explained to the participants who met the inclusion criteria. The participants voluntarily signed a written informed consent form before joining the study and were free to withdraw at any time without affecting their medical care. Informed consent was obtained from all participants.

## 3 RESULTS

In this study, the majority of the women were primiparous with a BMI above 25 and an anterior placental location. The obstetrical and clinical characteristics of the participants are shown (Table 1).

**Table 1:** Obstetrical and clinical characteristics of the participants.

Characteristic	*N* = 313
Age (M±SD)	30.54 ± 5.89
Gravidity *N* (%)	
1	112 (35.8)
<1	201 (64.2)
Parity *N* (%)	
Nulliparous	140 (44.7)
Primiparous	148 (47.3)
Multiparous	25 (8)
BMI *N* (%)	
>25	99 (31.6)
<25	187 (59.7)
Location of the placenta *N* (%)	
Anterior	138 (44.1)
Posterior	120 (38.3)
Fundal	23 (7.7)
Lateral	24 (7.7)
Low lying	5 (1.6)

Analyzing the correlation between GA based on ultrasound, showed a significant relationship with placental thickness (*p* = <0.0001) such that the correlation between placental thickness and current GA was 0.729, meaning that with increasing weekly GA, placental thickness increased by 72%, or 0.72. After analyzing the correlation of placental location with placental thickness, a significant relationship was found (*p* = 0.009). The correlation of placental thickness with placental location was *r* = 0.148. In posterior position, placental thickness increased by 14% or 0.14. The mean of placental thickness (29.49 ± 0.75) in the posterior part was greater than the thickness in the anterior part (26.94 ± 10.72). After analyzing the correlation between BMI and placental thickness, a significant relationship was also seen (*p* = 0.029). The correlation between placental thickness and BMI was *r* = 0.129, meaning that with increasing BMI group, placental thickness increased by 0.12 or 12% (Table 2).

**Table 2:** Investigating the relationship between the studied variables and the thickness of the placenta.

	Placental thickness	
	Type of test	*p*‐value
Gestational age (weeks)	Spearman correlation *R* = 0.729	<0.0001
Maternal age	Spearman correlation *R* = −0.116	0.040
Location of the placenta	Spearman correlation *R* = 0.148	0.009
Body mass index	Spearman correlation *R* = 0.129	0.029
Parity	Spearman correlation *R*= −0.046	0.420

There was a significant correlation between placental thickness and pregnancy trimester, particularly in the first (under 14 weeks) and second trimesters (Table 3).

**Table 3:** Comparison of placental thickness based on pregnancy trimester.

Trimester	Sample size	correlation	*p*‐value
First trimester	55	0.269	0.047
Second trimester	157	0.444	0.0001
Third trimester	101	0.088	0.381

Table 4 displays the mean and standard deviation and 95% confidence interval of placental thickness at different GAs based on a reliable LMP or ultrasound under 12 weeks.

**Table 4:** Average placental thickness for different ages of GA based on reliable LMP or ultrasound under 12 weeks.

Pregnancy week	Mean	Std. deviation	95% Confidence Interval for Mean	
			Lower Bound	Upper Bound
11.00	15.4838	3.96438	12.1694	18.7981
12.00	17.4480	2.88063	15.3873	19.5087
13.00	16.6142	3.20013	14.5809	18.6474
14.00	20.3161	5.35337	17.6539	22.9783
15.00	19.9960	7.23595	14.8197	25.1723
16.00	21.8550	4.71640	19.3418	24.3682
17.00	21.2478	5.26544	18.6293	23.8662
18.00	23.9636	7.39257	19.6952	28.2319
19.00	22.8400	5.33156	20.2703	25.4097
20.00	25.6830	6.59188	20.9675	30.3985
21.00	26.3233	4.67715	23.3516	29.2951
22.00	27.3800	5.98902	22.3731	32.3869
23.00	27.3929	9.04497	19.0277	35.7581
24.00	34.2629	10.03942	24.9779	43.5478
25.00	27.5854	7.85951	22.8359	32.3348
26.00	27.3500	4.21501	24.1101	30.5899
27.00	32.9323	7.64566	28.3121	37.5525
28.00	31.4278	8.71599	24.7281	38.1275
29.00	33.1643	5.77745	27.8210	38.5075
30.00	33.3567	9.16393	27.5342	39.1792
31.00	38.8540	8.47795	34.1591	43.5489
32.00	41.7469	9.48749	36.0137	47.4802
33.00	41.5415	11.84560	34.3833	48.6998
34.00	39.2673	10.00146	33.7287	44.8060
35.00	41.6700	11.81972	32.5846	50.7554
36.00	41.7813	7.92383	35.1568	48.4057
37.00	34.6238	5.83739	29.7436	39.5039
Total	28.6815	10.82707	27.4773	29.8856

Abbreviations: GA, gestational age; LMP, last menstrual period.

**Figure 1:**
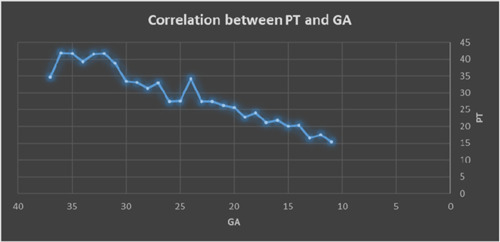
Correlation between PT (placental thicknesses) and GA (GA) table. GA, gestational age.

With increasing GA, placental thickness increases in a positive and linear manner. The increase in placental thickness is 72% or 0.72 with increasing gestational age, as shown in Figure 1.

## 4 DISCUSSION

Our analysis revealed a significant increase in PT with increasing GA during the first (under 14 weeks) and second trimesters. Additionally, there was a significant increase in PT in the posterior position of the placenta compared to the anterior position. Moreover, PT significantly increased with increasing maternal BMI.

The placenta is closely related to both the fetus and the mother, acting as a mirror that reflects their conditions.^22^ Thus, in this study, we investigated the relationship between PT and GA, placental position, and maternal BMI to examine and prevent potential risks for the fetus and assist physicians in delivery planning. Our results showed a positive and linear correlation between PT and GA, which could be due to two stages of placental growth during pregnancy: a phase of cellular proliferation lasting up to 36 weeks of gestation and a maturation phase of cellular hypertrophy from 36 weeks to term. The placenta must also undergo various changes to accommodate the metabolic demands of the embryo.^23^, ^24^ This finding is consistent with previous studies, such as Adhikari et al.,^17^ who observed that PT gradually increased from 11 mm at 11 weeks of gestation to 38.33 mm at the 40th week of pregnancy. Additionally, from 11 to 34 weeks of gestation, PT was approximately the same as GA, but after 35–40 weeks, PT was estimated as 1–2 mm less than GA. The other study conducted by Balakrishnan and Virudachalam^13^ concluded that measuring PT is an important parameter in estimating fetal age, especially during 25–35 weeks of gestation, during which PT complies closely with GA. Karthikeyan et al.^25^ concluded that PT has a linear relationship with GA. Several other studies also confirm this correlation.^5^, ^20^, ^26^ However, Ohagwu et al. showed that PT was 45.1 ± 6.4 mm at 39 weeks of gestation and could not explain why it was higher than in other studies. They speculated that PT may vary among races and be thicker in Negroes.^27^ Since all of the above studies are cross‐sectional, it is not possible to say that PT can be used as a reliable predictor of GA. Longitudinal studies involving multiple centers and large sample sizes are necessary to make a judgment on this result.

Our results also showed a significant relationship between placental location and PT, with thicker PT in the posterior position compared to the anterior position. This finding is consistent with some previous studies; such as Lee et al.^28^ who concluded that posterior placentals are approximately 0.7 cm thicker than anterior placentals. Additionally, Durnwald and Mercer^29^ showed that in second and third trimester of pregnancy, PT of posterior placentals was significantly greater than anterior placentals. In contrast, Hoddick et al.'s findings who found no relationships between placental l location and PT. This discrepancy may be due to technological limitations in ultrasonography in the past.^30^

In another part of the results, we observed that PT increased with maternal BMI. Our literature found only two studies that examined the effect of maternal obesity on PT.^29^, ^31^ Consistent with our studies, Farley et al.'s study showed a significant increase of 69% PT in obesity baboons.^31^ However, Durnwald and Mercer's study indicated that a BMI over 30 kg/m^2^ did not affect PT.^29^ In humans, maternal obesity may lead to various changes in the placenta, including: inflammation,^32^ increased villitis,^33^ macrophage infiltration,^34^ increased placental vascularity,^35^ and increased muscularity of placental vessels.^36^ In the present study, the increase in PT in obese mothers is likely due to mechanisms. Given the limited on the effect of maternal BMI on PT, further research during clinical research is needed to confirm or disprove this.

## 5 CONCLUSION

In conclusion, our study showed a significant increase in placental thickness with increasing GA in the first and second trimesters. Furthermore, placental thickness significantly increases in the placental posterior position and women with high BMI. We recommend that placental thickness be routinely measured during obstetric USGs.

## AUTHOR CONTRIBUTIONS

**Seyedeh Hajar Sharami**: Conceptualization; supervision; writing—review & editing. **Forozan Milani**: Data curation; methodology; writing—original draft. **Sima Fallah Arzpeyma**: Conceptualization; data curation; writing—review & editing. **Seyedeh Fatemeh Dalil Heirati**: Conceptualization; data curation; methodology; writing—original draft. **Zahra Pourhabibi**: Formal analysis; methodology; software.

## CONFLICTS OF INTEREST STATEMENT

The authors declare no conflicts of interest.

## TRANSPARENCY STATEMENT

The lead author Forozan Milani affirms that this manuscript is an honest, accurate, and transparent account of the study being reported; that no important aspects of the study have been omitted; and that any discrepancies from the study as planned (and, if relevant, registered) have been explained.

## Data Availability

Related data of this project are available upon request.
